# Changes in accelerometer-measured physical activity and self-reported leisure time physical activity from adolescence to young adulthood: a longitudinal cohort study from the Fit Futures Study

**DOI:** 10.1186/s12966-025-01799-4

**Published:** 2025-07-15

**Authors:** Tord Markussen Hammer, Jonas Johansson, Nina Emaus, Anne-Sofie Furberg, Luis Gracia-Marco, Bente Morseth, Ole Andreas Nilsen, Esther Ubago-Guisado, Dimitris Vlachopoulos, Marc Weitz, Elin Evensen, Tore Christoffersen

**Affiliations:** 1 School of Sports Sciences, Faculty of Health Sciences, UiT The Arctic University of Norway, Tromsø, Norway; 2 Department of Community Medicine, Faculty of Health Sciences, UiT The Arctic University of Norway, Tromsø, Norway; 3 Department of Health and Care Sciences, Faculty of Health Sciences, UiT The Arctic University of Norway, Tromsø, Norway; 4 Faculty of Health and Social Sciences, Molde University College, Molde, Norway; 5 Department of Microbiology and Infection Control, University Hospital of North-Norway, Tromsø, Norway; 6 Department of Physical Education and Sports, Faculty of Sport Sciences, Sport and Health University Research Institute (iMUDS), University of Granada, Granada, Spain; 7 Instituto de Investigación Biosanitaria, ibs.Granada, Granada, Spain; 8 Children’s Health and Exercise Research Centre, Public Health and Sport Sciences, University of Exeter Medical School, Exeter, UK; 9 Department of Computer Science, Faculty of Science and Technology, UiT The Arctic University of Norway, Tromsø, Norway; 10 Finnmark Hospital Trust, Alta, Norway

**Keywords:** Physical activity, Accelerometers, Self-report, Adolescence, Young adulthood, Sedentary time, Longitudinal study, Population-based cohort

## Abstract

**Background:**

Adolescence is associated with declining physical activity (PA) levels, and potential prevailing changes into young adulthood are indicated, but less explored. This study investigates longitudinal changes in PA from adolescence to young adulthood among males and females in a North Norwegian cohort.

**Methods:**

In the population-based Fit Futures Study, PA was assessed with both questionnaires (Saltin-Grimby Physical Activity Level Scale) and accelerometers (ActiGraph) at ages ~ 16 (n_self−report_=936; n_accelerometer_=674), ~ 18 (n_self−report_=808; n_accelerometer_=507), and ~ 27 (n_self−report_=648; n_accelerometer_=466). We used mixed effects models to analyze longitudinal changes in accelerometer-measured PA and sedentary time, alongside mixed effects multinomial logistic regression for changes in self-reported leisure time PA.

**Results:**

We observed a significant non-linear U-shaped trend in accelerometer-measured moderate-to-vigorous PA (MVPA) over time (*p* < 0.001), with an initial decline in minutes per day from age 16 (mean ± SD: 70.7 ± 25.2) to age 18 (62.3 ± 23.8), followed by an increase to age 27 (67.5 ± 30.4). At age 16, males exhibited higher MVPA than females. By age 18 and 27, MVPA levels were similar between sexes. Accelerometer-measured sedentary time decreased linearly across all three surveys (*p* = 0.002). We observed distributional shifts in self-reported leisure time PA over time: vigorously- and highly active proportions declined, while the moderately active proportion increased, and the proportion of sedentary participants remained stable (~ 20%). Compared to vigorously active, the odds of reporting sedentary (OR: 1.07, 95% CI: 1.03 to 1.11), moderately active (OR: 1.11, 95% CI: 1.07 to 1.15), and highly active (OR: 1.07, 95% CI: 1.03 to 1.11) increased with each year from age 16 (all *p* ≤ 0.001). Compared to moderately active, the odds of reporting other categories decreased over time (ORs: 0.92 to 0.96, all *p* ≤ 0.001).

**Conclusions:**

We observed non-linear changes in accelerometer-measured MVPA, indicating a U-shaped trend with a decline from 16 to 18 years, followed by an increase to age 27. Self-reported leisure time PA levels declined from adolescence to young adulthood, with decreasing proportions highly and vigorously active, while the proportion moderately active increased and the proportion of sedentary was unchanged. These results indicate that from adolescence to young adulthood, not all PA changes lead exclusively to increased sedentariness.

**Supplementary Information:**

The online version contains supplementary material available at 10.1186/s12966-025-01799-4.

## Background

Regular physical activity (PA) is associated with several health benefits [1], including improved cardiorespiratory fitness [2], more favorable levels of cardiometabolic risk factors [3], and enhancements in bone- [4] and mental health [5, 6]. Physical inactivity is a recognized risk factor for major non-communicable diseases [7], which represent a substantial economic burden globally [8]. Despite this, the prevalence of insufficient PA, defined as not meeting the WHO PA guidelines [9], is high and increasing among adults and adolescents, particularly in high-income countries [10, 11].

PA levels tend to decline throughout the lifespan, potentially beginning as early as 7–10 years of age [12, 13]. High PA levels in adolescence have been shown to predict sustained higher levels in adulthood [14–16]. At the very least, being physically active in adolescence may delay the onset of inactivity in adulthood [13]. Still, adolescence is marked by significant decreases in PA [17–24], with an estimated decline of approximately 7% per year in western high-income countries [25]. Generally, adolescent boys are more physically active than girls [11, 12, 17, 19–21, 26–28], yet they also experience greater reductions in PA during this period as they approach adulthood [25].

The transition from adolescence to adulthood is increasingly recognized as a critical period for health promotion [15, 29]. During this phase, PA levels may exhibit high variation [14, 18]. Although the decline in PA is found to continue in adulthood, it might do so at a slower rate than during adolescence [17], with less pronounced declines observed in females [28]. Moreover, it is indicated that PA changes may be non-linear further into adulthood [30]. However, most studies on PA changes from adolescence to young adulthood include measurements up to age 21, with very few studies extending beyond age 25 [17], and there is a scarcity of studies with more than two time points, leaving potential non-linearities of PA change largely unexplored.

Changes in PA have predominantly been investigated using self-reported measures [31], but these measures are more susceptible to recall and response bias than device-measured PA, and unable to capture absolute PA levels [32]. Accelerometer data may reveal different patterns of PA changes from adolescence to young adulthood compared to self-report measures, such as intensity distribution shifts [33] or relative stability [34]. Therefore, longitudinal study designs and more objective measures of PA (i.e. accelerometers) are needed [13, 15, 17, 35]. Still, self-reports can capture activity types not recorded by accelerometers (i.e. swimming), and activities outside the limited accelerometer wear time. Devices are also unable to contextualize or differentiate between PA domains, like occupational or leisure time [36, 37]. Combining device- and self-reported measurements could yield a more complete picture of PA behaviors [25, 38].

Therefore, this study aims to explore the changes in PA in adolescent girls and boys transitioning into young adulthood. Specifically, we analyze longitudinal PA changes from ages ~ 16 to ~ 18 and further to ~ 27 years, using both accelerometer-measured PA and self-reported leisure time PA.

## Methods

### Participants

We used data from the Fit Futures Study (FF), a population-based cohort study in Northern Norway which comprises three waves of data collection: FF1 (2010–2011), FF2 (2012–2013), and FF3 (2021–2022). The study targeted all upper-secondary schools located in the municipalities of Tromsø and Balsfjord. All first-year students, representing the 11th school year in the Norwegian educational system, were invited to participate in FF1. All participants from FF1 were invited to subsequent follow-up surveys, with 133 new students joining at FF2, and all previous participants were re-invited to FF3.

### Data collection

At FF1 and FF2, accelerometer measurements were collected during the school year, mostly between November and March, while assessments were evenly distributed throughout the year from September to August at FF3 (Additional Table 1). All participants were instructed to wear an accelerometer on their right hip for seven consecutive days, removing it only during sleep and activities involving water (e.g. showering, swimming, sauna) or activities with a high degree of physical interference (e.g. martial arts). Following consensus recommendations [39], accelerometer data were considered valid if there were at least four valid days with ≥ 10 h per day of wear-time, regardless of weekend or weekdays. At all three surveys, data was collected with an ActiGraph accelerometer (ActiGraph, LLC, Pensacola, USA), specifically the versions GT3X at FF1 and FF2, and the wGT3X-BT at FF3. These two generations of accelerometers provide equal metrics, including mean vector magnitude (VM) counts and when classified into activity intensities based on VM counts [40]. Raw accelerometer data were sampled at 30 Hz for FF1 and FF2, and at 100 Hz for FF3. For count-based metrics, the microprocessor embedded within the accelerometer performs a downsampling of the raw signal to 30 Hz regardless of original sampling frequency, ensuring comparability between sampling rates [41].

**Table 1:** Participant characteristics at ages 16-, 18-, and 27 years. The Fit Futures Study

	16 years (FF1)	18 years (FF2)	27 years (FF3)
Female	462 (49)	454 (56)	363 (56)
Male	474 (51)	354 (44)	285 (44)
Age, years	16.1 (0.4)	18.4 (0.7)	26.8 (0.8)
Height, cm	171.0 (8.9)	171.8 (9.4)	172.3 (9.2)
Weight, kg	65.6 (13.8)	68.8 (14.6)	77.1 (16.8)
BMI, kg/m^2^	22.4 (4.1)	23.2 (4.2)	25.9 (5.0)
Underweight	10 (1)	11 (1)	10 (2)
Normal weight	677 (73)	569 (74)	314 (49)
Overweight	171 (18)	125 (16)	204 (32)
Obesity	75 (8)	61 (8)	110 (17)
**Accelerometer PA**, n	674	507	466
Wear time, days	6.3 (1.4)	6.3 (1.4)	6.9 (1.5)
Wear time, min/day	842 (67)	823 (78)	794 (80)

Notes: Data on age, height, weight, BMI and accelerometer wear time/days are presented as means ± (standard deviations). Data on sex and BMI categories are presented as number of participants and percentages (%). Valid accelerometry data are presented as the number of participants. BMI categories for FF1 and FF2 are calculated according to the World Health Organization’s (WHO) age- and sex-specific cut-offs for ages 5–19. BMI categories for FF3 are calculated according to WHO standards for adults

To provide count-based metrices, the raw data were processed using the ActiLife software (ActiGraph, LLC, Pensacola; USA), employing the wear-time algorithm by Choi et al. [42]. Activity intensities were categorized using triaxial VM counts per minute (CPM) cut-points according to Peterson et al. [43] for sedentary behavior (1-149 VM CPM) and Sasaki et al. [44] for moderate PA (2690–6166 VM CPM), vigorous PA (6167–9642 VM CPM) and very vigorous PA (> 9642 VM CPM), effectively defining light PA as 150–2689 VM CPM. As the primary outcome, we analyzed the composite measure of moderate-to-vigorous PA (MVPA), which includes all movement at or above the moderate cut-point (≥ 2690 VM CPM). This represents higher-intensity PA. Furthermore, average daily CPM measurements are provided as an assessment of overall PA volume across the intensity spectrum.

At all three surveys, participants answered a questionnaire on health and lifestyle factors, where leisure time PA was measured using the “Saltin-Grimby Physical Activity Level Scale” (SGPALS). The SGPALS is a validated questionnaire that categorizes leisure time PA into four hierarchical and mutually exclusive categories [45, 46]. Participants were instructed to select the description that best represented their leisure time PA over the last year. The response options were described as:

1. Reading, watching TV, or other sedentary activity.
2. Walking, cycling, or other forms of exercise at least 4 h a week (including walking or cycling to place of work/school, shopping, Sunday-walking, etc.)
3. Participation in sports/training, heavy outdoor activities, snow clearing etc. at least 4 h a week.
4. Participation in hard training or sports competition regularly several times a week.

For our purposes in the current investigation, these categories have been assigned the following labels describing leisure time PA level; 1: *Sedentary*, 2: *Moderately active*, 3: *Highly active*, and 4: *Vigorously active*.

At all three waves of data collection, physical examinations were conducted by experienced technicians. Height and body weight were measured to the nearest 0.1 centimeter and 0.1 kg, respectively, wearing no shoes and light clothing on an automatic electronic scale (Jenix DS 102 stadiometer, Dong Sahn Jenix, Seoul, Korea).

Body mass index (BMI) was calculated as body weight in kilograms divided by the square of height in meters. For FF1 and FF2, BMI categories were defined using the World Health Organization’s (WHO) age- and sex-specific cut-offs and reference data for ages 5 to 19 years [47]. These categories included *Underweight* (<-2SD), *Normal weight* (-2SD to + 1SD) *Overweight* ( > + 1SD and ≤ + 2SD), and *Obesity* ( > + 2SD). For FF3, BMI was classified according to WHO standards for adults [48], with categories as follows: *Underweight* (< 18.5 kg/m^2^), *Normal weight* (18.5 to 24.9 kg/m^2^), *Overweight* (25.0 to 29.9 kg/m^2^), and *Obesity* (> 30 kg/m^2^).

### Statistical analyses

With normally distributed data, descriptive study population data were presented as means ± standard deviations (SD), or as number of participants and percentages. Two-sample t-tests with two-tailed *p*-values were used to analyze group mean differences in MVPA at each time point. We employed mixed effects models to analyze changes in accelerometer-measured PA and sedentary time from FF1 to FF2 to FF3. Time was operationalized as time elapsed from baseline, with FF1 set as year 0, FF2 as year 2, and FF3 as year 11. Wear time and month of accelerometer assessment were included as covariates to account for potential measurement variability. Models were fitted with random intercepts and random slopes at the participant level, allowing for individual variance in intercepts and rate of change. Furthermore, the models included a quadratic term for time to investigate potential non-linear patterns in PA development. To account for possible heteroscedasticity of residuals, models were fitted with robust standard errors. Adjusted estimated means for each time point were generated for sedentary time and activity categories using the adjusted models. Missing data were handled with the maximum likelihood estimation method of the mixed effects models. This allows for inclusion of all observations, including those for participants with incomplete data (i.e., those with measurements at only one or two surveys), under the missing at random assumption, in which missing data can be explained by the observed data [49]. All analyses were performed both stratified by sex and for the two sexes combined, incorporating a cross-product term between sex and time to explore interactions and provide a comprehensive understanding of the data.

To investigate change over time in self-reported leisure time PA, we used mixed effects multinomial logistic regression, estimated via maximum likelihood that allows participants with incomplete data to contribute to the analysis. Time was included as a fixed effect in the model (years elapsed from baseline), with results reported as Odds Ratios (OR) and 95% Confidence Intervals (CI). Random intercepts were included at the participant level to account for individual differences in baseline PA levels while evaluating the general trend in PA over time. The overall significance of temporal changes was assessed using likelihood ratio tests comparing models with and without the time variable.

To explore whether findings are biased by including data from participants with incomplete records (i.e. only participating at FF1), sensitivity analyses were conducted using only complete cases with valid measurements from all three surveys. Besides excluding the maximum likelihood estimation method, these mixed effects models were fitted identically as the main analyses, incorporating random intercepts, random slopes, a quadratic term for time, and with robust standard errors.

All statistical analyses were performed using Stata version 18 (StataCorp LLC, Texas, United States). The mixed effects multinomial logistic regression was performed with the user-written program gllamm (version 2.1.03). For all analyses, an alpha level of 0.05 was applied to determine statistical significance.

### Ethics

The Fit Futures Study is a longitudinal population-based health survey and was approved by the Norwegian Data Protection Authority and the Regional Committee for Medical and Health Research Ethics, North Norway (reference number: 2009/1282/ REK Nord) before initiation of the first wave of data collection in 2010. All participants provided written informed consent at all three surveys. Participants under 16 years of age at FF1 signed with additional written permission from their legal guardians. The present study was also approved by the Regional Committee for Medical and Health Research Ethics (reference number: 2023/684216/ REK Nord).

## Results

**Fig. 1:**
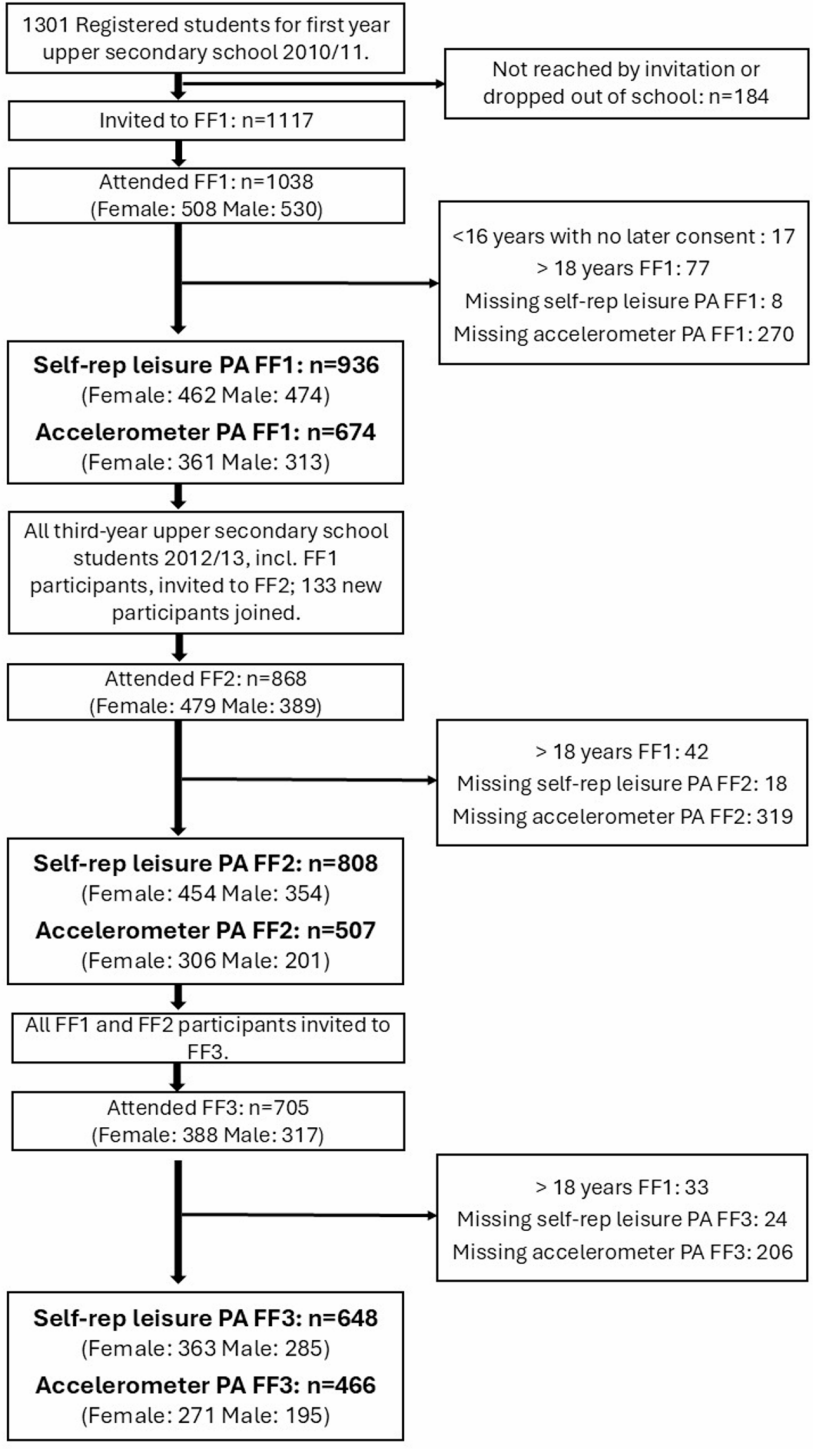
Flow chart of included participants

For this study, we included participants who were under 18 years old at FF1, who had completed the questionnaire on self-reported leisure time PA or had valid accelerometer data. The inclusion/exclusion process and resulting sample sizes are depicted as a flow chart in Fig. 1.

Participant characteristics for FF1, FF2, and FF3 are presented in Table 1, and sex stratified descriptives can be found in Additional Table 2. The mean (SD) age at attendance was 16.1 (± 0.4), 18.4 (± 0.7), and 26.8 (± 0.8) years at FF1, FF2, and FF3, respectively. Mean BMI increased for each survey and the combined proportion of participants categorized as overweight or obese increased from about 25% at ages 16 and 18 years, to nearly 50% at age 27 years. Accelerometer wear time and wear days were similar across all three surveys (Table 1).

**Table 2:** Accelerometer-measured physical activity and sedentary time, and self-reported leisure time physical activity levels at ages 16-, 18-, and 27 years. The Fit Futures Study

	16 years (FF1)	18 years (FF2)	27 years (FF3)
**Accelerometer PA**, n (% female)	674 (54)	507 (60)	466 (58)
Light, min/day	194.5 (46.5)	193.8 (53.3)	189.9 (58.5)
Moderate, min/day	60.8 (19.4)	54.1 (18.2)	58.7 (24.9)
Vigorous, min/day	8.1 (7.2)	6.4 (6.8)	7.7 (9.4)
Very vigorous, min/day	1.8 (3.0)	1.7 (6.6)	1.2 (3.0)
MVPA, min/day	70.7 (25.2)	62.3 (23.8)	67.5 (30.4)
CPM, counts/min	563 (179)	527 (173)	579 (217)
Sedentary, min/day	577.6 (70.5)	567.1 (80.0)	536.7 (84.4)
PA guideline compliance*	330 (49)	502 (99)	451 (97)
**Self-reported leisure time PA**, n (% female)	936 (49)	808 (56)	648 (56)
*Sedentary*	201 (21.5)	161 (19.9)	129 (19.9)
*Moderately active*	304 (32.5)	269 (33.3)	289 (44.6)
*Highly active*	243 (26.0)	230 (28.5)	151 (23.3)
*Vigorously active*	188 (20.0)	148 (18.3)	79 (12.2)

Notes: PA: Physical activity. MVPA: Moderate-to-vigorous PA. CPM: Counts per minute

Data on valid accelerometry and valid self-reported leisure time PA are presented as number of participants and percentage female. Data on accelerometer categories are presented as means ± (standard deviations). Data on PA guideline compliance and self-reported leisure time PA levels are presented as number of participants and (%)

*Meeting the World Health Organization’s PA guidelines,* defined as ≥ 60 min of moderate-to-vigorous PA (MVPA) per day for individuals under 18 years and ≥ 150 min of MVPA each week for individuals ≥ 18 years.*

**Fig. 2:**
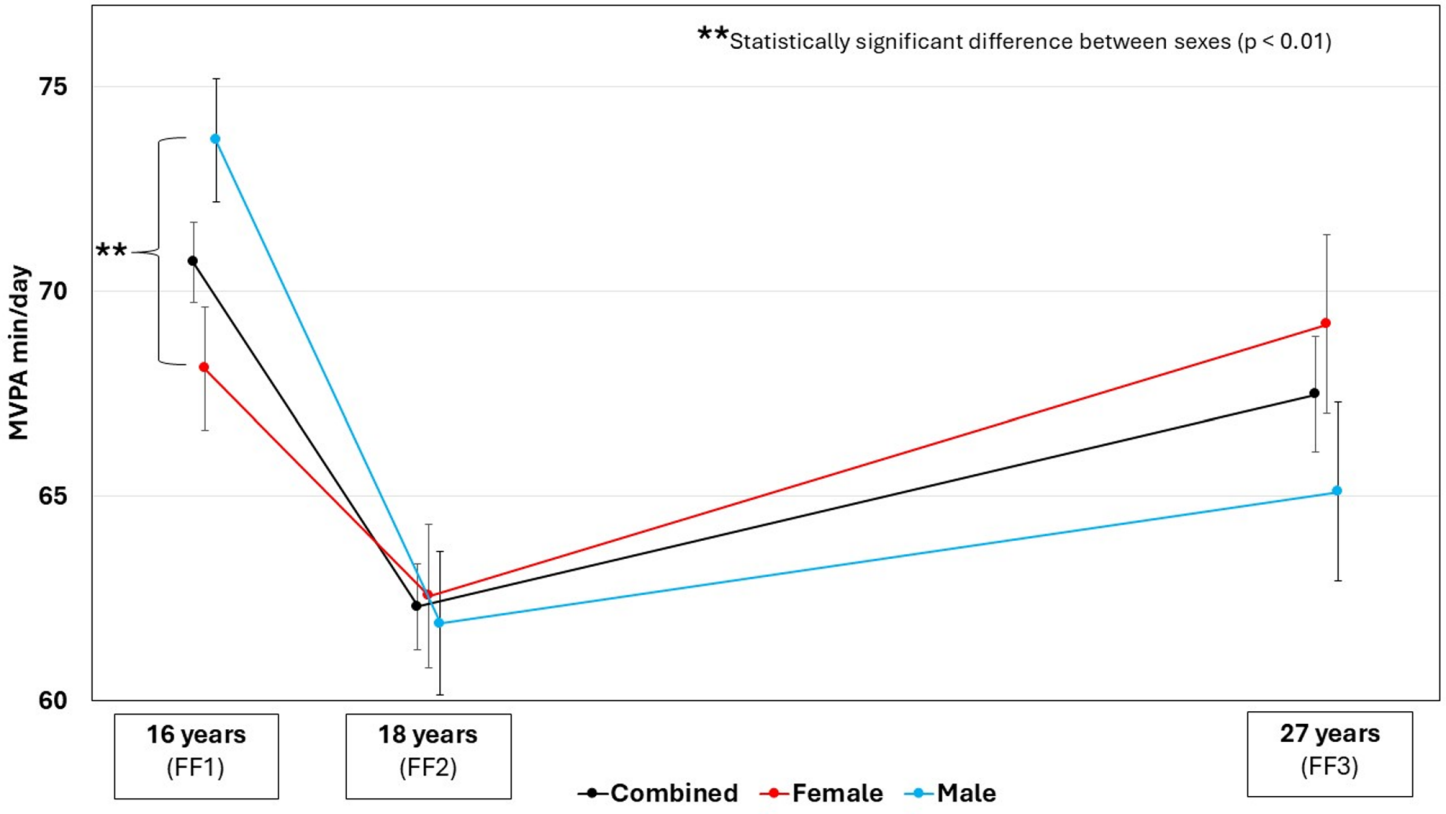
Longitudinal changes in accelerometer-measured moderate-to-vigorous physical activity (MVPA). The Fit Futures Study. Data are shown as mean with error bars as standard error

Mean minutes of MVPA per day decreased from age 16 (70.7 ± 25.2) to 18 (62.3 ± 23.8) and then increased to age 27 years (67.5 ± 30.4) (Table 2; Fig. 2) adjacent to the adjusted estimated means (Additional Table 3). Both sexes showed similar non-linear U-shaped patterns in MVPA, but females accumulated significantly less MVPA compared to males at age 16 years (*p* = 0.004), with 68.1 (± 23.5) min/day and 73.7 (± 26.6) min/day, respectively (Fig. 2 and Additional Table 4). At ages 18 and 27, mean MVPA-minutes per day were similar in females and males (*p* > 0.05). At age 16, 49% of participants met the PA guidelines for children and adolescents, while at age 18 there was nearly universal compliance with adult PA guidelines which remained at age 27. We observed a decline in accelerometer-measured sedentary time for each consecutive survey, but after adjustment, the estimated means display no change from 16 to 18 years followed by decline to 27 years (Additional Table 3).

**Table 3:** Effects of time on accelerometer-measured physical activity and sedentary time. The Fit Futures Study

	β	95% CI	*p*
**Light** (min/day)			
Time	0.71	0.20 to 1.22	0.007
Intercept	188.7	185.5 to 191.8	< 0.001
**Moderate** (min/day)			
Time	-2.98	-3.99 to -1.96	< 0.001
Time^2^	0.29	0.20 to 0.38	< 0.001
Intercept	59.01	57.65 to 60.37	< 0.001
**Vigorous** (min/day)			
Time	-0.72	-1.10 to -0.34	< 0.001
Time^2^	0.07	0.04 to 0.10	< 0.001
Intercept	7.73	7.21 to 8.26	< 0.001
**Very vigorous** (min/day)			
Time	-0.04	-0.07 to 0.01	0.012
Intercept	1.74	1.53 to 1.95	< 0.001
**MVPA** (min/day)			
Time	-3.67	-4.96 to -2.38	< 0.001
Time^2^	0.35	0.24 to 0.46	< 0.001
Intercept	68.45	66.69 to 70.20	< 0.001
**CPM**			
Time	-17.92	-27.29 to -8.55	< 0.001
Time^2^	1.87	1.06 to 2.68	< 0.001
Intercept	557.4	544.1 to 570.6	< 0.001
**Sedentary** (min/day)			
Time	-1.08	-1.77 to -0.39	0.002
Intercept	565.9	561.7 to 570.2	< 0.001

Notes: MVPA: Moderate-to-vigorous physical activity. CPM: Counts per minute

Models were adjusted for wear time and month of accelerometer assessment

The results from our mixed effects models, based on 1647 observations from 869 unique participants, showed a significant non-linear relationship between MVPA and time elapsed from baseline (Table 3). We observed a significant positive quadratic coefficient (*p* < 0.001) indicating that the U-shape is statistically significant. This non-linear relationship was consistent across sexes (Additional Table 5). There was a significant negative interaction term between sex and Time (*p* = 0.011) indicating that males had a steeper decline at baseline, while the significant positive interaction term between sex and Time^2^ (*p* = 0.048) suggests a more pronounced attenuation for males.

Mixed effect models (Table 3) for the separate intensity categories similarly show that non-linear relationships with time; an initial decline followed by a later increase, was found for moderate PA and vigorous PA, as well as CPM. For light PA we found a small linear increase, and for very vigorous PA, a small linear reduction in minutes per day from adolescence to young adulthood was found. We observed a linear decline in sedentary time from adolescence to young adulthood, but in the sex stratified analyses, this was only found for females (*p* < 0.001) and not males (*p* = 0.609).

We observed distributional shifts in self-reported leisure time PA levels over time, as the proportion of vigorously and highly active participants declined, while the proportion reporting a moderate activity level increased, and the proportion of sedentary participants remained stable (Table 2). Results from the likelihood ratio test confirmed that time was a statistically significant predictor of these changes (χ^2^ [3] = 34.75, *p* < 0.001). Our mixed effects multinomial logistic regression analysis of self-reported leisure time PA levels over time (*n* = 1070 individuals, 2392 observations) revealed significant shifts towards lower activity levels (Table 4). Compared with vigorously active, there was an increased odds for reporting sedentary (OR: 1.07, 95% CI: 1.03 to 1.11), moderately (OR: 1.11, 95% CI: 1.07 to 1.15), and highly active (OR: 1.07, 95% CI: 1.03 to 1.11) for each year passed from age 16 (all *p* ≤ 0.001). With moderately active as the reference category, the odds for reporting any other category decreased (ORs ranging from 0.92 to 0.96, all *p* ≤ 0.001). Compared with sedentary, the odds for reporting moderately active increased over time, while the odds for reporting vigorously active decreased over time. There was no non-linear pattern in the effect of time, and no significant interaction effects between sex and time for any of the categories.

**Table 4:** Effects of time (per one year) on self-reported leisure time physical activity levels. The Fit Futures Study

	OR	95% CI	*p*
**Sedentary (ref)**	1.00	Reference	-
Moderately active	1.03	1.00 to 1.06	0.021
Highly active	0.99	0.96 to 1.02	0.572
Vigorously active	0.96	0.93 to 0.99	0.018
**Moderately active (ref)**	1.00	Reference	-
Sedentary	0.96	0.93 to 0.98	0.001
Highly active	0.95	0.93 to 0.97	< 0.001
Vigorously active	0.92	0.89 to 0.95	< 0.001
**Highly active (ref)**	1.00	Reference	-
Sedentary	1.01	0.98 to 1.04	0.497
Moderately active	1.05	1.02 to 1.07	< 0.001
Vigorously active	0.97	0.94 to 0.99	0.050
**Vigorously active (ref)**	1.00	Reference	-
Sedentary	1.07	1.03 to 1.11	< 0.001
Moderately active	1.11	1.07 to 1.15	< 0.001
Highly active	1.07	1.03 to 1.11	0.001

Note: OR: Odds Ratio. 95% CI: 95% Confidence interval

The sensitivity analyses including only complete cases with valid accelerometer data for all three surveys counted 222 (60% female) participants. The results of this restricted analysis were consistent with those from our primary analysis. All key findings remained statistically significant, supporting the notion that allowing non-complete cases to contribute to the model does not bias our results (Additional Tables 6 and 7).

## Discussion

In this longitudinal study investigating changes in PA from ages 16 to 18 and further to 27 years we used accelerometer data from 869 participants and self-reported data from 1070 participants. The accelerometer analysis revealed non-linear, U-shaped changes in MVPA from adolescence to young adulthood. Initially, MVPA decreased from age 16 to 18, followed by an increase towards age 27, alleviating most of the earlier decline.

The longitudinal changes in self-reported leisure time PA indicated a shift towards moderate activity levels, with the odds of reporting vigorously active decreasing over time and the odds of reporting moderately active increasing versus all other levels. The odds of reporting being sedentary increased versus the vigorously active category, while it decreased versus moderately active. Overall, these results suggest a trend where individuals subjectively report their leisure time PA levels to decrease from age 16 to 27 years, which aligns well with previous research [17]. Importantly, this reported decline does not necessarily equate to increased sedentariness, as the proportion of participants reporting a sedentary leisure time PA level remained stable, while the proportion reporting a moderate leisure time PA level increased. This may indicate a shift in activity patterns rather than a mere reduction in overall leisure time activity, providing nuances to the understanding of how leisure time PA levels evolve during the transition from adolescence to young adulthood.

The observed decline in accelerometer-measured MVPA from age 16 to 18 broadly aligns with previously described PA patterns [17]. However, very few of the studies in the meta-analysis by Corder et al. [17] followed individuals beyond age 21. Our study expand these findings by showing that most of the accelerometer-measured MVPA-decrease rebounded, as average MVPA levels at age 27 years recovered to values similar to those at age 16. Additionally, the proportion of participants who fulfilled the WHO PA guidelines based on accelerometry was 49% at age 16, but compliance increased to 99% and 97% at ages 18 and 27, respectively. However, this discrepancy is primarily due to the differing guideline requirements between adults (150 min MVPA/week) versus adolescents (60 min MVPA/day) [9], highlighting the challenge in directly comparing compliance [38]. Our findings suggest that PA may not continuously decrease with age, despite the observed decline in late adolescence. Like the present study, the aforementioned meta-analysis also notes that the decline in PA from adolescence to young adulthood is less pronounced than during adolescence [17]. While the meta-analysis may have detected initial signs of a reversal in PA trends similar to our findings, it might also have been constrained by the short average follow-up period (3.4 years) and the completion of most studies by age 21.

Husøy et al. [34] also investigated accelerometer-measured PA longitudinally in a Norwegian cohort and found that MVPA was relatively stable from age 15 to 24. Our findings confirm this as the present study’s MVPA levels were similar at ages 16 and 27 years. Furthermore, we provide important nuance, as the measurements at age 18 years illustrated a significant, yet temporary, decline. Further research should investigate the stability in accelerometer-measured MVPA beyond 27 years of age.

Our findings of non-linear changes across the transition from adolescence to young adulthood could indicate that PA is influenced by the rapid and simultaneous biological, psychological and social changes typically associated with adolescence [50], along with challenges of maneuvering social transitions [51]. In Norway, age 18 represents some key transitions, such as entering legal adulthood, which may present new responsibilities, and attending the last year of upper secondary school leading to final exams and graduation. It is possible that these transitions have short term impact on PA levels. In light of our and others’ results [34], one could speculate that once such changes and transitions conclude or stabilize, PA levels may also stabilize, recover or even improve. Regardless, the period of young adulthood is a critical developmental period in life [52], and potential mechanisms underlying PA level changes should be further explored.

Our results show that males initially display greater MVPA but experience a greater reduction during adolescence compared to females, consistent with previous research [20, 21, 25–28]. Although boys were more active at age 16, the amount of MVPA evens out by ages 18 and 27. Similarly, Husøy et al. [34] found that females’ MVPA in young adulthood matches that of males, despite lower levels in adolescence. This suggests that while sex differences in PA exist during adolescence, they may diminish across the transition to young adulthood. However, this may be geographically and socially influenced, as studies from other regions report sustained sex differences in young adulthood, particularly in contexts of greater economic inequality [53]. As societal gender inequality has been found to amplify sex differences in PA [54], Norway’s high level of gender parity [55] may contribute to PA equality between sexes in young adulthood.

There is some discrepancy between the accelerometer measures, which show a non-linear pattern, and the self-reported measures, which indicate a steady decrease in leisure time PA over time. This may be due to the questionnaire’s explicit focus on leisure time PA, while accelerometers capture all movements regardless of context. Thus, individuals may engage in PA during work or school hours that is not captured by the questionnaire. Possibly, young adults experience less opportunity for leisure time PA than adolescents but able to acquire more PA in other domains. Another possible explanation for the discrepancy between measures is the phrasing of the questionnaire, which combines intensity and duration requirements. The moderately active response requires a minimum of four hours per week of “*walking*,* cycling*,* or other forms of exercise*”. Because the lower MVPA threshold (e.g. ≥2690 VM CPM) [44] corresponds to walking at about 5 km/h [56], individuals can engage in significant MVPA and still fall within this category. The highly active category requires at least four hours of *“sports/training”*, meaning individuals performing some high-intensity activity, may still report as moderately active if they do not meet the duration requirement. Additionally, the sedentary category’s description, *“Reading*,* watching TV*,* or other sedentary activity”*, sets a low bar, highlighting the broad range of the moderately active category. Thus, individuals may increase their accelerometer-measured MVPA but remain in the moderately active self-reported category. Generally, this aligns with previous literature, which indicates that PA questionnaires are less suited than accelerometry for estimating total PA volume [57].

In our study, accelerometer-measured sedentary time showed a linear decline across all three surveys for both sexes, which supports that PA changes at the end of adolescence are not solely towards adulthood inactivity. Two previous studies have assessed accelerometer-measured sedentary time longitudinally from adolescence to young adulthood [33, 34]. Our results are quite similar to those by Husøy et al. [34], which showed no change in sedentary time in a cohort of Norwegian adolescents. It is worth noting that PA and sedentary time varies between different countries and that Norwegian children have been reported to be more active than children in other European countries [12, 21]. In contrast to our results, Vanhelst et al. [33] showed a small ten-year increase in sedentary time from age ~ 15 years for participants in Belgium, France, Italy and Spain, and the difference in results might be partially explained by regional differences.

### Strengths and limitations

A key strength of the current study is the longitudinal cohort design, allowing registration of PA changes over time within the same individuals, enhancing the reliability and minimizing cohort effects typical in cross sectional designs. With data from three time-points, we were able to assess non-linearity. Furthermore, using both accelerometry and self-reports adds robustness, as the accelerometers mitigate recall bias and social desirability bias while capturing continuous PA intensity measurements. Our self-reported instrument, SGPALS, assesses the subjective perception of average leisure time PA in the last 12 months and might better capture activities that accelerometers often miss (e.g., swimming, cycling) and identify overall PA habits beyond the one-week accelerometer protocol. Together, these methods provide a comprehensive view of PA behaviors. Mixed effects models and multinomial logistic regression allowed for trend evaluation while accounting for baseline differences, and enabled inclusion of accelerometer data from 869 individuals and self-reports from 1070 participants, strengthening the findings. Another important strength is the population-based sampling (inviting all students starting upper secondary school in the study year) which provides a more representative baseline than convenience samples and strengthening external validity.

There are limitations to this study that should be noted. Throughout the study period (2010–2022) there was some participant attrition, particularly for the accelerometer measurements, which could be due to participants declining to wear an accelerometer, or due to lack of adherence to the protocol (i.e. not meeting the requirement of at least 4 days with 10 h of measurement). Our data suggests that the reduction in participants with valid accelerometer data is largely a function of overall study attrition rather than a specific issue with the accelerometer protocol. Selective compliance could potentially lead to biasing the sample towards more motivated, physically active, or health-conscious individuals. Our drop-out analysis (Additional file 2) revealed some differences between attendees and non-attendees, such as sex, smoking status and high school study program, but differences were less pronounced at the third survey wave. Although attrition and incomplete data present challenges, the use of mixed effects models with maximum likelihood estimation helps mitigate this impact. This approach, along with our sensitivity analysis of complete cases, enhances the robustness of our findings. Derived from a Norwegian cohort, these findings may reflect contextual factors specific to the setting. Therefore, generalizability beyond similar countries is unclear, but the population-based cohort design could support broader interpretation.

Caution is also advised when interpretating accelerometer-measured PA values, as they are derived from raw signals using specific algorithms and cut points, which can affect estimates. For instance, the data in this study has been previously analyzed with uniaxial values, a different wear-time algorithm and alternative cut points yielding different activity estimates [58]. These factors should be considered when evaluating durations and intensities and when comparing with other studies. However, the main objective in this study was to assess the longitudinal changes within individuals so most importantly the same procedure is applied to all times of measurement, and the cut points used are validated in a group of young adults [44].

## Conclusions

Accelerometer-measured MVPA showed a non-linear U-shaped pattern from adolescence to young adulthood in males and females, with a decline from 16 to 18 years of age, followed by an increase to age 27 years. Self-reported leisure time PA showed a general decline over the study period, with decreasing proportions of high and vigorous PA levels and increasing proportions reporting a moderately active PA level. This was parallel to no increase in the proportion of sedentary individuals. Taken together, these results might alleviate concerns for adulthood PA as individuals in the adolescence-young adult transition period do not exclusively move towards being more sedentary but possibly to activity at lower intensities, even increasing their MVPA following the initial adolescent decline.

## Electronic supplementary material

Below is the link to the electronic supplementary material.

**Supplementary Material 1**: **Additional file 1**: Additional Tables 1, 2, 3, 4, 5 and 6, and 7.

**Supplementary Material 2**: **Additional file 2**: Drop out analysis, Additional Tables 8 and 9.

**Supplementary Material 3**

## Data Availability

The data underlying the findings of this study are subject to ownership restrictions and are not publicly available but provided by the Fit Futures under license. Data can be made available upon reasonable request to the Fit Futures data and publication committee: fit.futures@uit.no.

## List of abbreviations

PA: Physical activity
WHO: World Health Organization
FF: Fit Futures Study
FF1: Fit Futures 1 (2010–2011)
FF2: Fit Futures 2 (2012–2013)
FF3: Fit Futures 3 (2021–2022)
SGPALS: Saltin-Grimby Physical Activity Level Scale
MVPA: Moderate-to-vigorous physical activity
VM: Vector Magnitude
CPM: Counts per minute
BMI: Body Mass Index
OR: Odds Ratio
CI: Confidence Interval
SD: Standard Deviation
