# Supplementary material for: Changes in accelerometer-measured physical activity and self-reported leisure time physical activity from adolescence to young adulthood: a longitudinal cohort study from the Fit Futures Study
Source: Int J Behav Nutr Phys Act. 2025 Jul 15;22:99. doi: 10.1186/s12966-025-01799-4 (PMC12265297; doi:10.1186/s12966-025-01799-4)
Supplement: Supplementary file 1 — Supplementary Material 1: Additional file 1: Additional Tables 1, 2, 3, 4, 5 and 6, and 7. [file 12966_2025_1799_MOESM1_ESM.docx]

**Additional Table 1:** Monthly distribution of accelerometer assessments at FF1, FF2 and FF3.

|  | **Sep** | **Oct** | **Nov** | **Dec** | **Jan** | **Feb** | **Mar** | **Apr** | **May** | **Jun** | **Jul** | **Aug** |
| --- | --- | --- | --- | --- | --- | --- | --- | --- | --- | --- | --- | --- |
| 16 years (FF1) | 35 (5%) | 59 (9%) | 96 (14%) | 47 (7%) | 131 (19%) | 139 (21%) | 105 (16%) | 62 (9%) |  |  |  |  |
| 18 years (FF2) |  |  | 93 (18%) | 69 (14%) | 165 (33%) | 140 (28%) | 30 (6%) | 1 (>1%) | 2 (>1%) | 7 (1%) |  |  |
| 27 years (FF3) | 50 (11%) | 44 (9%) | 34 (7%) | 26 (6%) | 12 (3%) | 10 (2%) | 44 (9%) | 59 (13%) | 60 (13%) | 63 (14%) | 21 (5%) | 43 (9%) |

Notes: Data are presented as number of participants and percentages (%).

**Additional Table 2:** Participant characteristics at ages 16-, 18-, and 27 years for females and males. The Fit Futures Study.

|  |  | **Female** |  |  |  | **Male** |  |
| --- | --- | --- | --- | --- | --- | --- | --- |
|  |  |  |  |  |  |  |  |
|  | **16 years** (FF1) | **18 years** (FF2) | **27 years** (FF3) |  | **16 years** (FF1) | **18 years** (FF2) | **27 years** (FF3) |
| n | 462 (49) | 454 (56) | 363 (56) |  | 474 (51) | 354 (44) | 285 (44) |
| Age, years | 16.1 (0.4) | 18.3 (0.8) | 26.8 (0.8) |  | 16.1 (0.4) | 18.3 (0.8) | 26.8 (0.8) |
| Height, cm | 164.9 (6.5) | 165.9 (6.5) | 166.5 (6.5) |  | 176.9 (6.7) | 179.4 (6.6) | 179.3 (6.8) |
| Weight, kg | 60.9 (11.5) | 63.5 (12.0) | 71.3 (14.8) |  | 70.0 (14.3) | 75.6 (14.8) | 84.2 (16.4) |
| BMI, kg/m^2^ | 22.4 (4.0) | 23.1 (4.2) | 25.7 (5.2) |  | 22.3 (4.1) | 23.3 (4.1) | 26.2 (4.7) |
| Underweight | 1 (0) | 1 (0) | 7 (2) |  | 9 (2) | 10 (3) | 3 (1) |
| Normal weight | 323 (77) | 337 (78) | 191 (54) |  | 323 (68) | 232 (70) | 123 (43) |
| Overweight | 73 (16) | 60 (14) | 93 (26) |  | 98 (21) | 65 (20) | 11 (39) |
| Obesity | 31 (7) | 35 (8) | 63 (18) |  | 44 (9) | 26 (8) | 47 (17) |
| Valid accelerometer, n | 361 (54) | 306 (60) | 271 (58) |  | 313 (46) | 201 (40) | 195 (42) |
| Wear time, days | 6.5 (1.3) | 6.4 (1.4) | 7.1 (1.5) |  | 6.1 (1.4) | 6.1 (1.5) | 6.8 (1.6) |
| Wear time, min/day | 843 (62) | 826 (74) | 797 (72) |  | 842 (73) | 819 (84) | 791 (90) |

Notes: Data on age, height, weight, BMI and accelerometer wear time/days are presented as means ± (standard deviations). Data on BMI categories are presented as number of participants and percentages (%). Data on valid accelerometry are presented as number of participants and percentage female and male). BMI categories for FF1 and FF2 are calculated according to the World Health Organization’s (WHO) age- and sex-specific cut-offs for ages 5-19. BMI categories for FF3 are calculated according to WHO standards for adults.

**Additional Table 3:** Adjusted estimated means for accelerometer-measured physical activity and sedentary time at ages 16-, 18-, and 27 years. The Fit Futures Study.

|  | **16 years** (FF1) | **18 years** (FF2) | **27 years** (FF3) |
| --- | --- | --- | --- |
|  |  |  |  |
| **Accelerometer PA**, n (% female) | 674 (54) | 507 (60) | 466 (58) |
| Light, min/day | 189 [186 to 192] | 194 [190 to 198] | 197 [192 to 202] |
| Moderate, min/day | 59.0 [57.7 to 60.4] | 54.2 [52.8 to 55.7] | 61.4 [59.2 to 63.6] |
| Vigorous, min/day | 7.7 [7.2 to 8.3] | 6.6 [6.0 to 7.1] | 8.2 [7.3 to 9.0] |
| Very vigorous, min/day | 1.7 [1.5 to 1.9] | 1.7 [1.1 to 2.2] | 1.3 [1.0 to 1.6] |
| MVPA, min/day | 68.4 [66.7 to 70.2] | 62.5 [60.6 to 64.4] | 70.8 [68.1 to 73.5] |
| CPM, counts/min | 557 [544 to 571] | 529 [515 to 543] | 587 [567 to 606] |
| Sedentary, min/day | 566 [562 to 570] | 567 [561 to 572] | 555 [548 to 561] |

Notes: PA: Physical activity. MVPA: Moderate-to-vigorous PA. CPM: Counts per minute.

Data on valid accelerometry are presented as number of participants and percentage female. Data on accelerometer categories are presented as means and [95% confidence intervals]. Models were adjusted for wear time and month of accelerometer assessment.

**Additional Table 4:** Sex stratified accelerometer-measured physical activity (PA) and sedentary time, and self-reported leisure time PA levels at ages 16-, 18-, and 27 years. The Fit Futures Study.

|  |  |  |  |  |  |  |  |  |  |  |  |
| --- | --- | --- | --- | --- | --- | --- | --- | --- | --- | --- | --- |
|  |  | **Female** |  |  |  | **Male** |  |  |  |  |  |
|  | **16 years** (FF1) | **18 years** (FF2) | **27 years** (FF3) |  | **16 years** (FF1) | **18 years** (FF2) | **27 years** (FF3) |  |  |  |  |
|  |  |  |  |  |  |  |  |  |  |  |  |
| **Accelerometer PA** | 361 (54) | 306 (60) | 271 (58) |  | 313 (46) | 201 (40) | 195 (42) |  |  |  |  |
| Light, min/day | 196.6 (41.6) | 198.4 (46.7) | 195.8 (51.3) |  | 192.0 (51.6) | 186.6 (61.5) | 181.9 (66.4) |  |  |  |  |
| Moderate, min/day | 59.5 (18.5) | 54.5 (17.0) | 60.1 (24.1) |  | 62.3 (20.4) | 53.6 (19.9) | 56.7 (25.8) |  |  |  |  |
| Vigorous, min/day | 6.9 (6.4) | 6.2 (7.0) | 8.1 (10.1) |  | 9.4 (7.9) | 6.8 (6.4) | 7.2 (8.3) |  |  |  |  |
| Very vigorous, min/day | 1.7 (2.8) | 1.9 (8.1) | 1.1 (2.3) |  | 2.0 (3.2) | 1.5 (3.1) | 1.2 (3.8) |  |  |  |  |
| MVPA, min/day | 68.1 (23.5) | 62.6 (23.0) | 69.2 (30.3) |  | 73.7 (26.6) | 61.9 (24.9) | 65.1 (30.4) |  |  |  |  |
| CPM, counts/min | 547 (161) | 531 (166) | 595 (209) |  | 582 (196) | 520 (182) | 556 (226) |  |  |  |  |
| Sedentary, min/day | 578.4 (62.1) | 565.0 (75.8) | 531.6 (75.7) |  | 576.7 (79.1) | 570.3 (86.2) | 543.9 (95.0) |  |  |  |  |
| PA guideline compliance* | 162 (45) | 303 (99) | 267 (99) |  | 168 (54) | 199 (99) | 184 (94) |  |  |  |  |
| **Self-reported leisure time PA** | 462 (49) | 454 (56) | 363 (56) |  | 474 (51) | 354 (44) | 285 (44) |  |  |  |  |
| *Sedentary* | 64 (13.9) | 65 (14.3) | 57 (15.7) |  | 137 (28.9) | 96 (27.1) | 72 (25.2) |  |  |  |  |
| *Moderately active* | 184 (39.8) | 192 (42.3) | 185 (51.0) |  | 120 (25.3) | 77 (21.8) | 104 (36.5) |  |  |  |  |
| *Highly active* | 137 (29.7) | 135 (29.7) | 85 (23.4) |  | 106 (22.4) | 95 (26.8) | 66 (23.2) |  |  |  |  |
| *Vigorously active* | 77 (16.7) | 62 (13.7) | 36 (9.9) |  | 111 (23.4) | 86 (24.3) | 43 (15.1) |  |  |  |  |

Notes: PA: Physical activity. MVPA: Moderate-to-vigorous PA. CPM: Counts per minute.

Data on valid accelerometry and valid self-reported leisure time PA are presented as number of participants and (% female/male). Data for accelerometer categories are presented as means ± (standard deviations). Data on PA guideline compliance and self-reported leisure time PA are presented as number of participants and percentage (%).

**Meeting the World Health Organization’s PA guidelines, defined as ≥60 minutes of moderate-to-vigorous PA (MVPA) per day for individuals under 18 years and ≥150 minutes of MVPA each week for individuals ≥ 18 years.*

**Additional Table 5:** Sex stratified effects of time on accelerometer-measured physical activity and sedentary time. The Fit Futures Study.

|  | **Female** | | |  | **Male** | |  | |
| --- | --- | --- | --- | --- | --- | --- | --- | --- |
|  | **β** | **95% CI** | **p** |  | **β** | **95% CI** | **p** |  |
| **Light** (min/day) |  |  |  |  |  |  |  |  |
| Time | 0.87 | 0.25 to 1.50 | 0.006 |  | 0.47 | -0.36 to 1.31 | 0.269 |  |
| Intercept | 191.2 | 187.3 to 195.1 | <0.001 |  | 186.0 | 180.9 to 191.0 | <0.001 |  |
| **Moderate** (min/day) |  |  |  |  |  |  |  |  |
| Time | -2.24 | -3.40 to -1.09 | <0.001 |  | -3.87 | -5.67 to -2.07 | <0.001 |  |
| Time^2^ | 0.24 | 0.14 to 0.34 | <0.001 |  | 0.34 | 0.19 to 0.50 | <0.001 |  |
| Intercept | 57.75 | 56.07 to 59.43 | <0.001 |  | 60.48* | 58.30 to 62.65 | <0.001 |  |
| **Vigorous** (min/day) |  |  |  |  |  |  |  |  |
| Time | -0.25 | -0.74 to 0.24 | 0.320 |  | -1.13** | -1.67 to -0.60 | <0.001 |  |
| Time^2^ | 0.04 | -0.002 to 0.08 | 0.061 |  | 0.09* | 0.04 to 0.13 | <0.001 |  |
| Intercept | 6.47 | 5.89 to 7.05 | <0.001 |  | 9.19*** | 8.33 to 10.05 | <0.001 |  |
| **Very vigorous** (min/day) |  |  |  |  |  |  |  |  |
| Time | -0.04 | -0.07 to 0.002 | 0.066 |  | -0.06 | -0.11 to -0.01 | 0.026 |  |
| Intercept | 1.55 | 1.29 to 1.80 | <0.001 |  | 2.01 | 1.64 to 2.38 | <0.001 |  |
| **MVPA** (min/day) |  |  |  |  |  |  |  |  |
| Time | -2.21 | -3.74 to -0.68 | 0.005 |  | -5.33* | -7.52 to -3.13 | <0.001 |  |
| Time^2^ | 0.26 | 0.12 to 0.39 | <0.001 |  | 0.46 | 0.27 to 0.65 | <0.001 |  |
| Intercept | 65.71 | 63.63 to 67.79 | <0.001 |  | 71.60** | 68.74 to 74.47 | <0.001 |  |
| **CPM** |  |  |  |  |  |  |  |  |
| Time | -5.83 | -16.62 to 4.96 | 0.290 |  | -33.00** | -49.53 to -16.48 | <0.001 |  |
| Time^2^ | 1.05 | 0.10 to 1.99 | 0.029 |  | 2.87* | 1.45 to 4.30 | <0.001 |  |
| Intercept | 539.4 | 524.1 to 554.6 | <0.001 |  | 578.3** | 556.1 to 500.4 | <0.001 |  |
| **Sedentary** (min/day) |  |  |  |  |  |  |  |  |
| Time | -1.62 | -2.45 to -0.79 | <0.001 |  | -0.30* | -1.45 to 0.85 | 0.609 |  |
| Intercept | 567.3 | 562.2 to 572.4 | <0.001 |  | 564.0 | 557.1 to 571.0 | <0.001 |  |

Notes: MVPA: Moderate-to-vigorous physical activity. CPM: Counts per minute.

Models were adjusted for wear time and month of accelerometer assessment.

*** Statistically significant difference between sexes (<0.001)

** Statistically significant difference between sexes (<0.01)

* Statistically significant difference between sexes (<0.05)

**Sensitivity analyses:**

**Additional Table 6:** Mean (SD) moderate-to-vigorous physical activity at FF1, FF2, and FF3 for participants with valid accelerometer data at all surveys:

|  | **Combined** (n = 222) | | |  | **Female** (n =134) | | |  | **Male** (n = 88) | | |
| --- | --- | --- | --- | --- | --- | --- | --- | --- | --- | --- | --- |
|  | **16 years** (FF1) | **18 years** (FF2) | **27 years** (FF3) |  | **16 years** (FF1) | **18 years** (FF2) | **27 years** (FF3) |  | **16 years** (FF1) | **18 years** (FF2) | **27 years** (FF3) |
| MVPA, min/day (±) | 69.6 (24.1) | 63.5 (25.4) | 68.8 (31.7) |  | 69.5 (22.3) | 65.1 (26.4) | 70.4 (33.7) |  | 69.8 (26.7) | 61.0 (23.5) | 66.5 (±28.4) |

Note: MVPA: Moderate-to-vigorous physical activity.

**Additional Table 7:** Mixed effects model results for effect of time on moderate-to-vigorous physical activity, counts per minute and sedentary time, including only complete cases.

|  | **Combined** (n = 222) | | |  | **Female** (n = 134) | | |  | **Male** (n = 88) | | |
| --- | --- | --- | --- | --- | --- | --- | --- | --- | --- | --- | --- |
|  | **β** | **p** | **95% CI** |  | **β** | **p** | **95% CI** |  | **β** | **p** | **95% CI** |
| **MVPA** (min/day) |  |  |  |  |  |  |  |  |  |  |  |
| Time | -2.96 | 0.002 | -4.80 to -1.12 |  | -2.05 | 0.078 | -4.34 to 0.23 |  | -3.97 | 0.010 | -6.97 to -0.97 |
| Time^2^ | 0.31 | <0.001 | 0.15 to 0.47 |  | 0.24 | 0.018 | 0.04 to 0.44 |  | 0.38 | 0.004 | 0.12 to 0.63 |
| (Intercept) | 67.18 | <0.001 | 64.22 to 70.13 |  | 67.10 | <0.001 | 63.71 to 70.49 |  | 67.26 | <0.001 | 61.91 to 72.61 |
| **CPM** |  |  |  |  |  |  |  |  |  |  |  |
| Time | -15.20 | 0.022 | -28.21 to -2.19 |  | 5.13 | 0.005 | 1.51 to 8.75 |  | -19.80 | 0.074 | -41.49 to 1.90 |
| Time^2^ | 1.63 | 0.005 | 0.50 to 2.76 |  | - | - | - |  | 2.06 | 0.031 | 0.19 to 3.93 |
| (Intercept) | 553.6 | <0.001 | 531.2 to 575.9 |  | 541.2 | <0.001 | 514.3 to 568.2 |  | 544.0 | <0.001 | 504.2 to 583.8 |
| **Sedentary** (min/day) |  |  |  |  |  |  |  |  |  |  |  |
| Time | -3.76 | <0.001 | -4.92 to -2.60 |  | -0.87 | 0.185 | -2.16 to 0.42 |  | -1.34 | 0.100 | -2.93 to 0.26 |
| (Intercept) | 581.44 | <0.001 | 572.6 to 590.3 |  | 568.8 | <0.001 | 559.7 to 577.8 |  | 571.2 | <0.001 | 558.5 to 583.9 |

Note: MVPA: Moderate-to-vigorous physical activity. CPM: Counts per minute.

Models were adjusted for wear time and month of accelerometer assessment.
