# Supplementary material for: Changes in accelerometer-measured physical activity and self-reported leisure time physical activity from adolescence to young adulthood: a longitudinal cohort study from the Fit Futures Study
Source: Int J Behav Nutr Phys Act. 2025 Jul 15;22:99. doi: 10.1186/s12966-025-01799-4 (PMC12265297; doi:10.1186/s12966-025-01799-4)
Supplement: Supplementary file 2 — Supplementary Material 2: Additional file 2: Drop out analysis, Additional Tables 8 and 9. [file 12966_2025_1799_MOESM2_ESM.docx]

**Additional file 2:** Drop out analysis.

To assess potential differences between the analyzed sample, defined as participants with valid data (attendees), and those not included, defined as participants without valid data (non-attendees), at each survey, we compared baseline characteristics (sex, BMI, PA, smoking frequency, alcohol frequency, and high school study program). Attendance statuses were defined separately for accelerometer-measured PA and self-reported leisure time PA based on data validity criteria, and for each survey wave. Continuous variables were analyzed using two-sample t-tests with two-tailed significance testing, while categorical variables were analyzed using chi-square tests. Analyses were conducted for each of the three surveys.

***Attendance defined by valid accelerometer data:***

16 years (FF1): Males were more likely non-attendees than females (p < 0.001). Non-attendees were more often sometime smokers (p < 0.001) and enrolled in vocational high school programs (p < 0.001) compared to attendees. More participants with lower self-reported leisure time PA did not provide valid accelerometer data. No differences were observed for BMI.

18 years (FF2): Males were more likely non-attendees than females (p=0.005). Non-attendees tended to smoke more often (p=0.037) and be enrolled in vocational high school programs (p < 0.001). No significant differences were observed for BMI or MVPA.

27 years (FF3): Attendees were more likely to be non-smokers at baseline (p=0.028). Students enrolled in vocational high school programs were less likely to provide valid accelerometer data (p=0.012). No significant differences were observed for sex, BMI, or MVPA.

***Attendance defined by valid self-reported leisure time PA:***

16 years (FF1): Due to the small number of non-attendees (n = 8), no significant differences were observed between attendees and non-attendees for any baseline characteristics.

18 years (FF2): Attendees were more likely to be non-smokers (p = 0.002). No significant differences were observed for sex, BMI, accelerometer-measured PA, self-reported leisure time PA, or high school program.

27 years (FF3): Students in vocational high school programs were less likely to attend (p=0.050). No significant differences were observed for sex, BMI, accelerometer-measured PA, self-reported leisure time PA, or smoking status.

**Additional Table 8:** Comparison of baseline characteristics between attendees (valid accelerometry) and non-attendees (non-valid accelerometry) for the three surveys FF1, FF2, and FF2.

|  | **16 years (FF1)** | | | **18 years (FF2)** | | | **27 years (FF3)** | | |
| --- | --- | --- | --- | --- | --- | --- | --- | --- | --- |
| **Variable FF1** | Valid accelerometry | Non-valid accelerometry | p-value | Valid accelerometry | Non-valid accelerometry | p-value | Valid accelerometry | Non-valid accelerometry | p-value |
| n | 674 | 270 |  | 418 | 279 |  | 420 | 188 |  |
| Sex |  |  |  |  |  |  |  |  |  |
| Female | 361 (54%) | 103 (38%) | <0.001 | 247 (59%) | 135 (48%) | 0.005 | 235 (56%) | 90 (48%) | 0.065 |
| Male | 313 (46%) | 167 (62%) |  | 171 (41%) | 144 (52%) |  | 185 (44%) | 98 (52%) |  |
| BMI | 22.4 | 22.4 | 0.969 | 22.2 | 22.3 | 0.893 | 22.3 | 22.3 | 0.885 |
| MVPA, min/day |  |  |  | 69.8 | 76.3 | 0.058 | 71.8 | 70.1 | 0.508 |
| Self-reported PA |  |  |  |  |  |  |  |  |  |
| Sedentary | 126 (19%) | 75 (28%) | 0.013 | 73 (18%) | 58 (21%) | 0.037 | 69 (17%) | 48 (26%) | 0.055 |
| Moderately active | 227 (34%) | 77 (29%) |  | 149 (36%) | 82 (30%) |  | 140 (33%) | 56 (30%) |  |
| Highly active | 182 (27%) | 61 (23%) |  | 121 (29%) | 67 (24%) |  | 114 (27%) | 47 (25%) |  |
| Vigorously active | 137 (20%) | 51 (19%) |  | 74 (18%) | 69 (25%) |  | 95 (23%) | 34 (18%) |  |
| Smoking |  |  |  |  |  |  |  |  |  |
| Non-smoker | 552 (82%) | 179 (68%) | <0.001 | 349 (84%) | 209 (76%) | 0.037 | 349 (84%) | 138 (74%) | 0.028 |
| Sometimes | 95 (14%) | 77 (29%) |  | 59 (14%) | 55 (20%) |  | 60 (14%) | 40 (22%) |  |
| Daily | 23 (3%) | 8 (3%) |  | 9 (2%) | 11 (4%) |  | 8 (2%) | 7 (4%) |  |
| Alcohol |  |  |  |  |  |  |  |  |  |
| Never | 205 (31%) | 57 (22%) | 0.001 | 134 (32%) | 74 (27%) | 0.013 | 118 (28%) | 58 (31%) | 0.191 |
| ≤Once/month | 285 (42%) | 106 (40%) |  | 184 (44%) | 109 (39%) |  | 186 (45%) | 68 (37%) |  |
| >Once/month | 181 (27%) | 100 (38%) |  | 98 (24%) | 93 (34%) |  | 113 (27%) | 59 (32%) |  |
| High school program |  |  |  |  |  |  |  |  |  |
| General | 310 (46%) | 78 (29%) | <0.001 | 228 (54%) | 97 (35%) | <0.001 | 209 (50%) | 77 (41%) | 0.012 |
| Sports | 76 (11%) | 28 (10%) |  | 36 (9%) | 44 (16%) |  | 52 (12%) | 16 (8%) |  |
| Vocational | 288 (43%) | 164 (61%) |  | 154 (37%) | 138 (49%) |  | 159 (38%) | 95 (51%) |  |

**Additional Table 9:** Comparison of baseline characteristics between attendees (valid self-reported leisure time physical activity) and non-attendees (non-valid self-reported leisure time physical activity) for the three surveys FF1, FF2, and FF2.

|  | **16 years (FF1)** | | | **18 years (FF2)** | | | **27 years (FF3)** | | |
| --- | --- | --- | --- | --- | --- | --- | --- | --- | --- |
| **Variable FF1** | Valid self-report | Non-valid self-report | p-value | Valid self-report | Non-valid self-report | p-value | Valid self-report | Non-valid self-report | p-value |
| n | 936 | 8 |  | 680 | 17 |  | 576 | 32 |  |
| Sex |  |  |  |  |  |  |  |  |  |
| Female | 462 (49%) | 2 (25%) | 0.170 | 376 (55%) | 6 (35%) | 0.102 | 311 (54%) | 14 (44%) | 0.258 |
| Male | 474 (51%) | 6 (75%) |  | 304 (45%) | 11 (65%) |  | 265 (46%) | 18 (56%) |  |
| BMI | 22.3 | 22.7 | 0.823 | 22.2 | 22.8 | 0.529 | 22.3 | 21.9 | 0.601 |
| MVPA, min/day | 70.7 | 67.9 | 0.876 | 71.7 | 78.8 | 0.338 | 71.3 | 73.4 | 0.686 |
| Self-reported PA |  |  |  |  |  |  |  |  |  |
| Sedentary |  |  |  | 127 (19%) | 4 (24%) | 0.681 | 108 (19%) | 9 (30%) | 0.445 |
| Moderately active |  |  |  | 226 (33%) | 5 (29%) |  | 189 (33%) | 7 (23%) |  |
| Highly active |  |  |  | 185 (27%) | 3 (18%) |  | 153 (27%) | 8 (27%) |  |
| Vigorously active |  |  |  | 138 (20%) | 5 (29%) |  | 123 (21%) | 6 (20%) |  |
| Smoking |  |  |  |  |  |  |  |  |  |
| Non-smoker |  |  |  | 549 (81%) | 9 (53%) | 0.002 | 464 (81%) | 23 (77%) | 0.320 |
| Sometimes |  |  |  | 106 (16%) | 8 (47%) |  | 95 (17%) | 5 (17%) |  |
| Daily |  |  |  | 20 (3%) | 0 (0%) |  | 13 (2%) | 2 (7 %) |  |
| Alcohol |  |  |  |  |  |  |  |  |  |
| Never |  |  |  | 203 (30%) | 5 (30%) | 0.748 | 165 (29%) | 11 (37%) | 0.626 |
| ≤Once/month |  |  |  | 287 (43%) | 6 (35%) |  | 242 (42%) | 12 (40%) |  |
| >Once/month |  |  |  | 185 (27%) | 6 (35%) |  | 165 (29%) | 7 (23%) |  |
| High school program |  |  |  |  |  |  |  |  |  |
| General | 387 (41%) | 1 (13%) | 0.077 | 319 (47%) | 6 (35%) | 0.342 | 276 (48%) | 10 (31%) | 0.050 |
| Sports | 104 (11%) | 0 (0%) |  | 79 (12%) | 1 (6%) |  | 66 (11%) | 2 (6%) |  |
| Vocational | 445 (48%) | 7 (87%) |  | 282 (41%) | 10 (59%) |  | 234 (41%) | 20 (63%) |  |
